# Scanning Electron Microscope: A New Potential Tool to Replace Gram Staining for Microbe Identification in Blood Cultures

**DOI:** 10.3390/microorganisms9061170

**Published:** 2021-05-28

**Authors:** Gabriel Haddad, Sara Bellali, Tatsuki Takakura, Anthony Fontanini, Yusuke Ominami, Jacques Bou Khalil, Didier Raoult

**Affiliations:** 1 Institut Hospitalo-Universitaire Méditerranée Infection 19-21 Boulevard Jean Moulin, 13005 Marseille, France; gabrielhaddad92@gmail.com (G.H.); sarabellali@hotmail.fr (S.B.); fontanini.anthony@gmail.com (A.F.); 2 Aix-Marseille Université, IRD, APHM, MEPHI, 27 Boulevard Jean Moulin, 13005 Marseille, France; 3 Hitachi High-Tech Corporation, Analytical & Medical Solution Business Group, 882 Ichige, Hitachinaka-shi 312-8504, Ibaraki, Japan; tatsuki.takakura.yk@hitachi-hightech.com; 4 Hitachi High-Tech Corporation, Nanotechnology Solutions Business Group, Toranomon Hills Business Tower, 1-17-1 Toranomon, Minato-ku, Tokyo 105-6409, Japan; yusuke.ominami.ay@hitachi-hightech.com

**Keywords:** scanning electron microscopy, gram, MALDI-TOF, blood culture, rapid test, microbe identification

## Abstract

Blood culture is currently the most commonly used method for diagnosing sepsis and bloodstream infections. However, the long turn-around-time to achieve microbe identification remains a major concern for clinical microbiology laboratories. Gram staining for preliminary identification remains the gold standard. We developed a new rapid strategy using a tabletop scanning electron microscope (SEM) and compared its performance with Gram staining for the detection of micro-organisms and preliminary identification directly from blood cultures. We first optimised the sample preparation for twelve samples simultaneously, saving time on imaging. In this work, SEM proved its ability to identify bacteria and yeasts in morphotypes up to the genus level in some cases. We blindly tested 1075 blood cultures and compared our results to the Gram staining preliminary identification, with MALDI-TOF/MS as a reference. This method presents major advantages such as a fast microbe identification, within an hour of the blood culture being detected positive, low preparation costs, and data traceability. This SEM identification strategy can be developed into an automated assay from the sample preparation, micrograph acquisition, and identification process. This strategy could revolutionise urgent microbiological diagnosis of infectious diseases.

## 1. Introduction

The most common method of diagnosing sepsis remains blood culture [1,2]. Nevertheless, rapid pathogen identification is a key component in the microbiological diagnosis [3,4]. The time-to-results has a significant and direct clinical impact and an impact on the prognosis of patients [5,6]. Currently, once a blood culture is detected positive, a smear is Gram stained for a first bacterial identification that most likely influences the treatment choice within the recommended timeframe [7,8]. This allows the treatment to be adjusted towards better antibiotic stewardship [9,10]. However, Gram staining results are not always reliable due to multiple factors, such as inter-operator variability, false staining for some species, etc. [11,12,13]. Within the last decade, insufficient identifications were switched to culture and eventually relied on other high accuracy methods for microbe identification in blood cultures such as matrix-assisted laser desorption/ionization time-of-flight mass spectrometry (MALDI TOF/MS) [14], molecular biology [15,16,17], multiplex PCR assays, specific hybridization assays, or microarrays [18,19,20,21]. Nevertheless, none of these methods, even though some of them are more costly, present a turn-around time that allows for rapid preliminary identification from the blood culture in the way Gram staining does. The search for new tools and strategies that can improve, automate, and accelerate a more specific and rapid preliminary identification continues to be a hot topic in bloodstream infection management [22,23,24].

Recently, a new generation of table-top scanning electron microscopes (SEM) has enabled the acquisition of micrographs more rapidly in comparison with other electron microscopes. Here, we evaluated the routine use of these user-friendly SEMs, that could potentially replace optical microscopes in our laboratory. Thus, we developed a strategy and a potential tool for rapid preliminary microbe identification directly from blood cultures that are identified as positive by the BacT/Alert automated microbial detection system (BioMérieux Clinical Diagnostics, Lyon, France) using SEM. Subsequently, we blindly tested 1075 blood cultures and analysed the accuracy, the expertise required, the identification information acquired, and the time taken to identify the micro-organism for the newly developed strategy and compared the results to those of conventional Gram staining preliminary identification.

## 2. Methods

### 2.1. Sample Selection

#### 2.1.1. Artificial Blood Cultures for the Developmental Stage

A selection of the most common bacteria found in blood cultures was collected from the Collection de Souches de l’Unité des Rickettsies (CSUR, WDCM 875) (Table S1, Figure S1). Briefly, bacteria were harvested from fresh cultures, inoculated into BioMérieux blood culture bottles (BacT/ALERT-FA-Plus, BioMérieux, Lyon, France), and incubated for 24 h at 37 °C to simulate clinical growth conditions.

#### 2.1.2. Clinical Blood Cultures for the Proof of Concept and Validation Stage

Nine hundred ninety-nine randomly selected blood cultures from the *Institut Hospitalo-Universitaire Méditerranée Infection* (IHU) clinical bacteriology unit were used for the developmental stage and proof of concept. As a negative control, virgin blood cultures were tested to assess the presence of structures that might affect the results. The final analysis was then blindly applied to a new series of 1075 randomly selected blood cultures. This study was approved by the IHU Ethics Committee (n°2018-016).

### 2.2. Sample Preparation Optimisation

**Figure 1:**
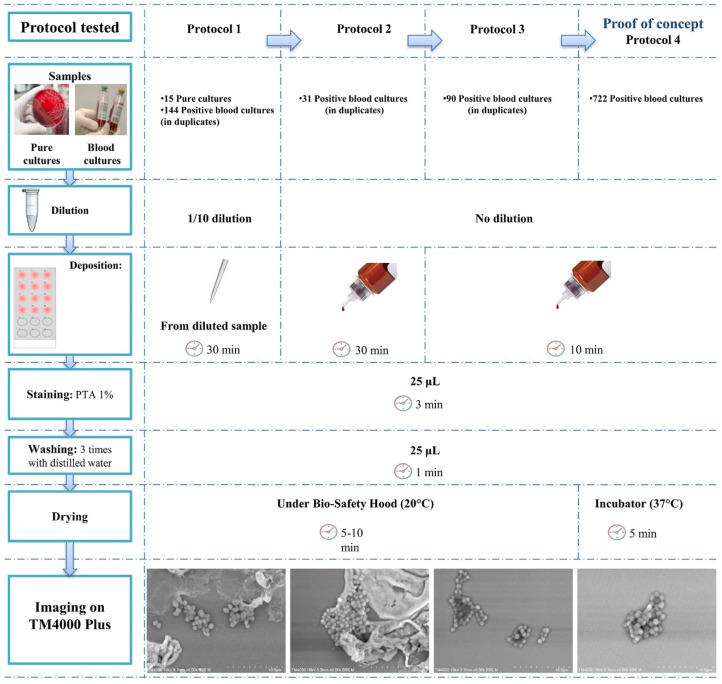
Optimisation of the sample preparation for imaging micro-organisms of 12 positive blood cultures. Reducing the sample preparation time was crucial since we aimed for early and fast identification of the microbes. This protocol was optimised and adapted after many modifications. Protocol 4 showed the best yield for image acquisition and analysis.

First, we tested multiple stains including phosphotungstic acid (PTA) (Sigma-Aldrich, St. Louis, MO, USA), osmium tetroxide, and molybdate to select the most suitable stain for our analysis. We then tested four protocols to find the sample preparation conditions for the best image quality and resolution (Figure 1**)**. We set and tested the preliminary protocol on artificial and clinical blood cultures. We optimised the protocol to shorten the overall sample preparation time. We considered the sample dilution, the incubation time on slide, the sample deposition, and the drying process. The final protocol (Figure 1. Protocol 4) was applied inside a moist chamber and on 12-well Poly-L-lysine coated slides (Sigma-Aldrich, UK—Immuno-Cell International, Antwerp, Belgium). Blood cultures were directly deposited in the wells using airway needles and incubated for ten minutes at room temperature (~25 °C). The excess liquid was then replaced by 1% PTA solution (pH 7.4) for three minutes before it was discarded. All wells were then rinsed three times for one minute each using sterile water and dried at 37 °C for five minutes. We calculated the cost for preparing the 12 samples using this optimised protocol as shown in Table S2.

### 2.3. Micrograph Acquisition

Micrographs were manually recorded using Hitachi’s TM4000 Plus tabletop SEM. The accelerating voltage was 10 kV, and magnifications ranged between 250× and 5000×. A newly developed software coupled with the TM4000 Plus enabled the automatic screening and acquisition of six micrographs for twelve samples within 25 min. When more investigations were needed for deeper morphological description, micrographs were recorded using Hitachi’s SU5000 SEM, aiming for higher magnifications and resolution. Low vacuum conditions were used, and single cells were targeted. The accelerating voltage of the SEM was 10 kV and magnifications ranged between 5000× and 15,000×.

### 2.4. Post-Acquisition Analysis

#### 2.4.1. Identification Based on Bacterial Morphology and Size

Analysis of the bacterial morphology with regards to shape and size were investigated on all blood cultures. We based our identification criteria on the microbial shapes that are the basics of fundamental microbiology [7,25]. We also identified specific features capable of differentiating between different microbes. Some criteria were known in optical microscopy; however, they require expertise to be determined on regular optical microscopes. Width and length measurements were made using Image-J [26]; 50 to 300 bacteria were measured. All mono-microbial blood cultures according to culture were categorised depending on their shape to classify the identified microbes into groups up to the genus level according to specific features. Poly-microbial blood cultures according to culture-based identification were analysed separately, as various aspects should be considered for screening and micrographs acquisition before the identification step.

#### 2.4.2. Cell Wall Analysis Based on Image Contrast

We determined the cell wall nature using SEM, comparing the bacterial brightness to the background. Analysis of the acquired micrographs was performed using Image-J software [26]. For cocci and bacilli, we used either histogram or line profiling for the pixel grayscale analysis.

### 2.5. Comparison to Routine Microbial Identification

The SEM identifications were compared to the routine clinical microbiology laboratory results. All the blood cultures were Gram stained (Aerospray Gram Slide Stainer) and observed by optical microscopy. In order to evaluate the inter-operator variability for Gram staining, results were compared for 650 slides analysed by experienced and inexperienced operators.

As a reference method, colonies from subcultures were also identified using MALDI TOF/MS (Microflex, Bruker Daltonics, Bremen, Germany) as previously described [27]. If no microbes were identified, the sub-culture incubation time was increased for ten days before confirming the absence of microbes. MALDI TOF/MS identification served as a reference in this study. When all methods used were inconclusive, discordances were resolved by partial 16S rRNA gene molecular sequencing.

We calculated the sensitivity, specificity, positive and negative predictive values, and the accuracy of our newly developed identification strategy with MALDI-TOF/MS as a gold standard.

## 3. Results

### 3.1. Sample Preparation Optimization for SEM

**Figure 2:**
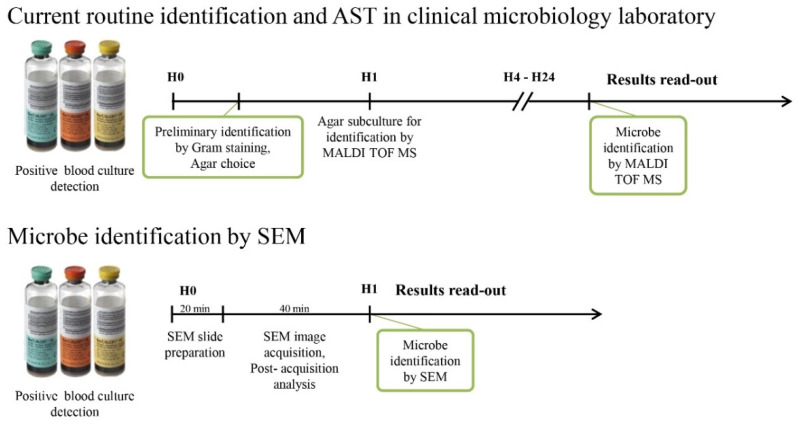
Timelines comparing routinely used methods for SEM identification. (**upper**) Gram staining starting from the positive blood culture, an essential step in redirecting the choice of the agar medium for the subculture. MALDI TOF MS identification is then applied to the colonies grown after 4–24 h of incubation. (**lower**) SEM identification starting from the positive blood culture through the identification of the microbe within one hour following the simple SEM strategy we developed.

No prior dilution of the sample was needed; therefore, the direct deposition from the blood culture reduced the sample deposition step to a minimum. When we reduced the incubation time on the slide, fewer aggregates and blood culture components were visible. Drying at 37 °C led to a faster drying process and had no effect on the quality of results. Therefore, protocol 4 (Figure 1) proved to be the optimal sample preparation protocol, giving the best compromise between image quality and sample preparation time. The choice of the 12-well slides allowed us to load twelve samples at once into the SEM chamber, hence the vacuum was performed once for twelve samples, reducing the acquisition time. The time from blood culture to microbe identification by SEM was thus one hour for twelve blood cultures (Figure 2). This time-to-results is comparable to that of the Gram staining for the same 12 blood cultures; however, this tool gives more insight into the microbes, allowing for a more specific preliminary identification, with data traceability. This method does not require a high budget after acquisition of the instrument, since the budget for the sample preparation of twelve samples is reduced by one third (0.83 EUR versus 2.39 EUR using SEM and Gram staining, respectively, Table S2).

### 3.2. Post-Acquisition Analysis

**Figure 3:**
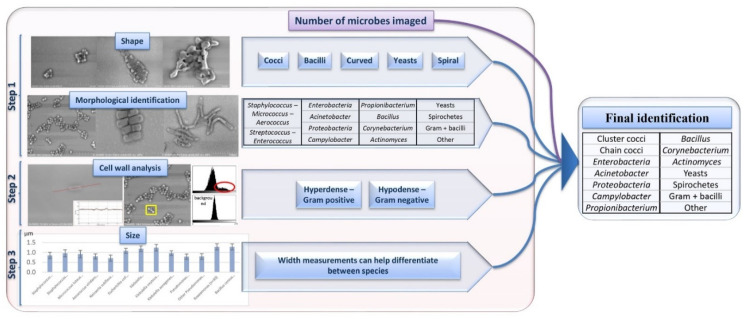
Merging steps 1, 2, and 3 allowed us to build a robust analysis for microbe identification.

We treated different aspects of the acquired micrographs to develop a robust analytical tool for microbe identification, including identification of the shape, disposition, size, and cell wall (Figure 3).

#### 3.2.1. Step 1: Identification Based on Microbial Morphology by SEM

**Figure 4:**
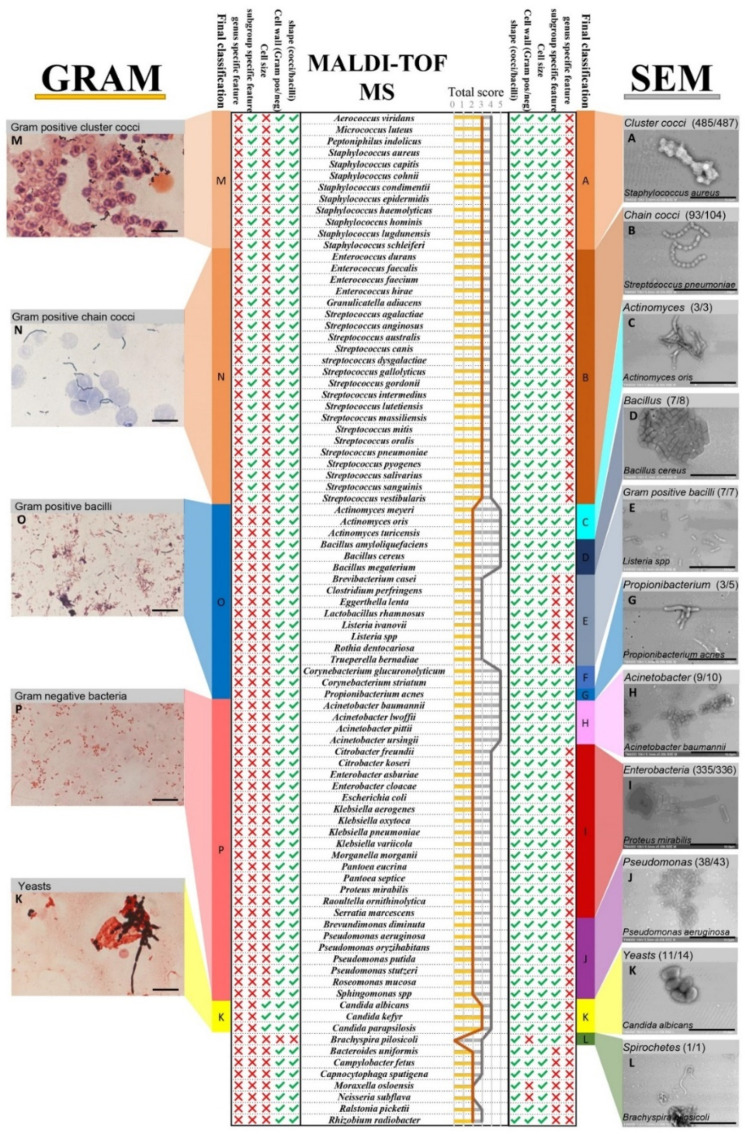
Microbe classification and identification criteria acquired using Gram staining versus SEM with MALDI-TOF/MS as a reference. Identification information (shape, cell wall, cell size, subgroup-specific feature, genus-specific feature) acquired for each of the species is indicated with a tick mark, missing information is indicated with a red cross. The total Scheme 100. artificial blood cultures inoculated with various *Acinetobacter* sp. and *Enterobacteria*. We were able to blindly identify 82% of the *Acinetobacter* according to the presence of these structures.

Bacterial morphology was a major factor in fast microbe identification (Figure 4). We were able to differentiate the micro-organisms on the micrographs first by their shape: cocci, bacilli, spirals, and others. We then clustered them according to their arrangement (chains, clusters, grouped by two). Aiming to differentiate the microbes more specifically, we defined specific features and associated them with various groups of micro-organisms. First, we were able to differentiate the cocci-shaped bacteria according to their arrangement. Cocci that arranged in clusters belonged to the group of *Staphylococcus* sp., *Micrococcus* sp., *Aerococcus* sp., and *Neisseria* sp. We confused *Neisseria* and *Staphylococcus* as they both presented grape-like shapes. Cocci arranged in chains were mainly *Enterococcus* sp., *Streptococcus* sp., and *Granulicatella* sp. For bacilli-shaped cells, the bacterial borders were the differential criteria: the square-shaped bacilli were identified as *Bacillus* sp., while *Propionibacterium* sp. were seen as boomerang shaped bacteria, *Actinomyces* sp. were arranged in networks, and other Gram-positive bacilli were defined by the presence of spores or the chain disposition.

We correlated each of these morphotypes to a group of bacteria (Figure 4). We were able to correctly classify 86.7% of the microbes according to the MALD-TOF/MS identification.

#### 3.2.2. Step 2: Cell Wall Analysis by SEM Based on Image Contrast

The PTA stain enabled us to differentiate between both cocci and bacilli according to their contrast compared to the micrograph background. Gram-positive bacteria had a contrast brighter than that of the background in the centre, showing increased brightness in the middle of the bacteria on the linear greyscale profile and a second peak on the greyscale histograms (Figure S2a,c). For the Gram-negative bacteria, the contrast was darker or comparable to that of the background, and the results were validated on the greyscale histograms and linear profiles (Figure S2b,d). When applied on the micrographs of 726 blood culture, we correctly classified 86% of the microbes judging by their contrast, according to the MALD-TOF/MS identification.

#### 3.2.3. Step 3: Identification Based on Microbial Size by SEM

*Proteobacteria* and *Enterobacteria* species presented comparable morphologies and similar greyscale profiles. To balance out these discordances, we analysed the bacterial width of these microbes. We found that the diameter of the *Enterobacteria* we measured was stable (1 µm) between the different species (*E. coli*, *Klebsiella* sp.), whereas the *Pseudomonas* sp. isolates were thinner (0.7 µm) and the *Bacillus* sp. isolates had wider diameters (1.2 µm). In addition, *Neisseria* sp. were smaller than *Staphylococcus* sp. but the sizes overlapped (Figure S3). Yeasts were considerably larger in size than bacteria, which helped to easily discern them.

### 3.3. SEM Compared to MALDI-TOF/MS Reference Identification

**Figure 5:**
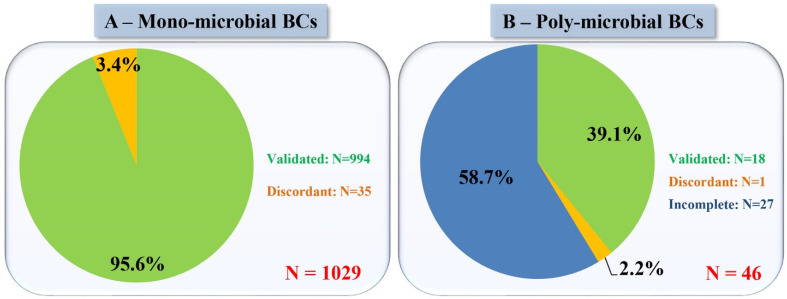
SEM identification results compared to those of MALDI-TOF/MS as a reference method in (**A**) mono-microbial blood cultures and (**B**) poly-microbial blood cultures. Validated: same identification results; Discordant: different identification results; Incomplete: Routine methods showed more microbes than did SEM.

Once all the data had been collected, we combined the validated criteria. All the information was compiled in order to establish a final robust classification strategy for direct SEM microbe identification, which was applied to 1029 mono-microbial blood culture micrographs (Figure 4). We managed to correctly classify 994 blood cultures (96.60%) of the analysed microbes with the SEM classification according to the MALDI-TOF/MS identification (Figure 5A). SEM misclassifications represented 35 blood cultures (3.4%). The most common discordance for most groups were dark smudges that were observed in 14 of the blood cultures (1.2%). This phenomenon may be caused by the destruction of bacteria, the low number of microbes present in the imaged sample, or the operator issues related to sample screening. Of the 21 remaining misidentifications, some species (*Bacteroides uniformis*, *Capnocytophaga sputigena*, *Eggerthella lenta*, *Moraxella osloensis*, *Ralstonia picketii*, *Escherichia coli*, *Pseudomonas* sp., *Acinetobacter* sp.) were identified by SEM in terms of shape, cell wall, and cell size, although not classified in any of the SEM groups. For example, *Pseudomonas* sp. and *Acinetobacter* sp. were classified as *Enterobacteria*; however, all were classified as Gram-negative bacilli with SEM.

### 3.4. SEM vs. Gram Staining

#### 3.4.1. Case of Blood Cultures Positive for Cocci

Gram staining enabled the differentiation of Gram-positive cocci arranged in clusters and in chains with high sensitivity (99.79% and 95.19%, respectively). Similarly, SEM enabled the differentiation of these two groups of bacteria with a higher sensitivity for the cluster cocci than the chain cocci (99.59% and 89.42%, respectively), with five blood cultures (0.49%) positive for chain cocci that were classified as cluster cocci (Figure S4c,d). All performance characteristics for both methods with culture-based identification as a reference are detailed in Table 1.

#### 3.4.2. Case of Blood Cultures Positive for Bacilli

For rod-shaped bacteria, Gram staining enabled the identification of Gram-negative bacilli with a sensitivity of 99.49%, while SEM sensitivity was 99.23% for this group. However, SEM proved to be efficiently able to separate the *Enterobacteria*, *Proteobacteria,* and *Acinetobacter* sp. within this same group of bacteria. Moreover, when Gram staining detected the Gram-positive bacilli with a sensitivity of 82.61%, the SEM method was sensitive to 86.96%. Likewise, using SEM, we were able to distinguish *Bacillus* sp., *Propionibacterium* sp., *Actinomyces* sp., and other Gram-positive bacilli. All performance characteristics for both methods with culture-based identification as a reference are detailed in Table 2.

#### 3.4.3. Case of Blood Cultures Positive for Other Microorganisms

Furthermore, the differentiation of yeasts is based on their size for both methods with a high accuracy. Interestingly, one blood culture which was detected as positive by the automate showed no microbes on the Gram slide and no growth on agar plates for ten days. SEM detected spirochetes (Figure 4L) that were identified after 16S rRNA gene sequencing as *Brachyspira pilosicoli*. Performance characteristics are detailed in Table 3.

#### 3.4.4. Case of Negative Blood Cultures and Poly-Microbial Blood Cultures

Negative blood cultures imaged by SEM had no visible structures that were similar in size or shape to any known microbes. SEM also made it possible to detect the presence of multiple microbes in a blood culture. In total, 46 poly-microbial blood cultures of the 1075 blood cultures were analysed separately, considering the number of microbes detected by SEM and their identification using our strategy. In fact, for 18 poly-microbial blood cultures, all existing microbes were correctly identified. However, for 27 of them, only imaged microbes were correctly identified based on the criteria we defined above. In total, in 97.8% of the poly-microbial blood cultures, at least one of the organisms present was correctly identified; however, the full pathogen identification was incomplete as not all micro-organisms were detected by SEM (Figure 5B). The problem here is related to the screening of the sample. A fairly large portion of the sample must be screened for the presence of multiple micro-organisms. Manual imaging creates an operator-dependent issue, once image acquisition is automated, screening related problems would be diminished.

### 3.5. Operator-Dependant Variability

Furthermore, when evaluating the inter-operator variability for Gram staining of 650 blood cultures, a total of 4% (26 blood cultures) presented divergent results between operators with different experience levels in reading Gram stain results. For the same 650 blood cultures, different results for different operators represented 1.38% (nine blood cultures) using SEM. Discrepancies between operators in reading SEM results could be resolved with data traceability for the micrographs.

## 4. Discussion

In this study, we evaluated in parallel SEM and Gram preliminary identifications on blood cultures that had been detected as positive, with MALDI-TOF/MS colony identification from subcultures as the gold standard reference. Several improvements were made during this work, reducing the SEM sample preparation time to 20 min and using 12-well slides to save time on imaging. Once the micro-organisms were detected, several morphological elements were gradually added to identify the microbe up to the genus level (shape, disposition of the microbes, diameter, the presence of specific structures, etc.). This new classification method enables more information from the bacterial morphology to be accessed for a more specific preliminary identification than that of the Gram staining (Figure 3). SEM enabled us to highlight the ultrastructure of the micro-organisms, giving more insight on their identification. We were able to differentiate five groups (*Bacillus* sp., *Propionibacterium* sp., *Actinomyces* sp., *Corynebacterium* sp., and other Gram-positive bacilli) within the bacteria classified as Gram-positive and rod-shaped using Gram staining. This method also helped to distinguish three groups within the bacteria classified as Gram-negative rods by Gram staining, to define a specific trait for detecting *Acinetobacter* sp., and to draw a distinction between *Enterobacteria* and *Proteobacteria*. On the clinical level, a preliminary identification as soon as the blood culture is detected as positive can potentially influence the course of antibiotic treatment [28]. The importance of such a rapid preliminary identification strategy relies on the redirection or essential adjustments to the antibiotic regimen, the need to start antibiotic treatment, or to take other immediate measures before the culture results become available [8].

Moreover, with the increase of the microbiological workload in clinical laboratories, automation is potentially conceivable for such strategies and is necessary to reduce the time-to-results in the case of blood cultures [29]. This type of strategy will lead to the creation of an image database and the development of a software using machine learning, allowing for the automatic identification of the objects observed. In the long term, this would replace the technical and microbiological knowledge necessary to be able to identify the bacteria with the commonly-used staining methods such as Gram and other dyes used for microorganisms [13,30]. A major part of the cost for our method comes from the purchase of a TM4000 Plus SEM (50 k EUR). One TM4000 Plus SEM can perform the SEM imaging of roughly 10,000 twelve-well slide per year, with a machine lifetime of 10 years. Therefore, the cost for one blood culture analysis by SEM can be estimated to 0.042 EUR per sample. For the Gram staining, the optical microscope cost is reduced by ten (5 k EUR). Even though the TM4000 Plus is more costly, the consumables cost for large scale analyses remains reduced compared to that of Gram staining. Gram staining also has manual labour requirements for Gram slides preparations and an increase in the read-out of results that lead to higher costs. After automation and standardisation of sample preparations and the identification processes, SEM could optimise workflows and reduce labour costs, leading to a fully automated pipeline for preliminary microbe identification.

For the past three years, we have been evaluating the various possible front-end applications and processes for this new generation of SEMs and their practical implementation in microbiological diagnosis. They present many advantages in terms of low cost, ease-of-use, speed of results, and the ability to observe a variety of clinical samples. Thus, SEM could advantageously replace optical microscopes and Gram staining in the preliminary identification of bacteria in blood cultures or other body fluids. We have already developed other applications for these SEMs in the rapid detection of respiratory viruses [31], rapid antibiotic susceptibility testing of bacteria [32], detection of bacteria in endocarditic cardiac valve vegetations [33], and identification of several parasites. These various uses of SEM will shift the paradigm of the early diagnosis of infectious diseases, especially in critical cases that have a direct effect on the management of bloodstream infections, the treatment choice, and the patients’ prognosis [15,34,35].

## Figures and Tables

**Table 1:** Performance characteristics for cocci-shaped bacterial identification using SEM vs. Gram staining with culture-based MALDI-TOF/MS identification as a reference.

Group Identified	Prevalence	Method	Sensitivity (95% CI)	Specificity (95% CI)	PPV (95% CI) *	NPV (95% CI) *	Accuracy (95% CI) *
**Cluster cocci**	47.33%	GRAM	99.79%	99.45%	99.39%	99.81%	99.61%
			(98.86% to 99.99%)	(98.39% to 99.89%)	(98.13% to 99.80%)	(98.70% to 99.97%)	(99.01% to 99.89%)
		SEM	99.59%	99.44%	99.38%	99.63%	99.51%
			(98.52% to 99.95%)	(98.38% to 99.88%)	(98.11% to 99.80%)	(98.54% to 99.91%)	(98.86% to 99.84%)
**Chain cocci**	10.11%	GRAM	95.19%	99.89%	99%	99.46%	99.42%
			(89.14% to 98.42%)	(99.40% to 100%)	(93.31% to 99.86%)	(98.74% to 99.77%)	(98.74% to 99.79%)
		SEM	89.42% (81.86% to 94.60%)	100%	100%	98.82% (97.96% to 99.32%)	98.93% (98.09% to 99.46%)
**Gram-negative cocci**	0.1%	GRAM	100%	100%	100%	100%	100% (99.64% to 100%)
		SEM	0%	100%	0%	99.9% (99.90% to 99.90%)	99.9% (99.45% to 100%)

PPV: Positive Predictive Value; NPV: Negative Predictive Value; (*) These values are dependent on the prevalence.

**Table 2:** Performance characteristics for bacilli-shaped bacterial identification using SEM vs. Gram staining with culture-based MALDI-TOF/MS identification as a reference.

Group Identified	Prevalence	Method	Sensitivity (95% CI)	Specificity (95% CI)	PPV (95% CI) *	NPV (95% CI) *	Accuracy (95% CI) *
**Gram-negative bacilli**	37.8%	GRAM	99.49%	99.22%	98.72%	99.69%	99.32%
			(98.16% to 99.94%)	(98.19% to 99.75%)	(97.00% to 99.46%)	(98.76% to 99.92%)	(98.60% to 99.73%)
		SEM	99.23%	98.75%	97.97%	99.53%	98.93%
			(97.76% to 99.84%)	(97.55% to 99.46%)	(96.04% to 98.97%)	(98.56% to 99.85%)	(98.10% to 99.47%)
*Enterobacteria*	32.65%	SEM	99.7%	99.13%	98.24%	99.85%	99.32%
			(98.35% to 99.99%)	(98.13% to 99.68%)	(96.18% to 99.20%)	(98.98% to 99.98%)	(98.60% to 99.73%)
*Proteobacteria*	4.18%	SEM	88.37%	99.9%	97.44%	99.49%	99.42%
			(74.92% to 96.11%)	(99.44% to 100%)	(84.24% to 99.63%)	(98.86% to 99.78%)	(98.74% to 99.79%)
*Acinetobacter* sp.	0.97%	SEM	90%	99.9%	89.98%	99.9%	99.81%
			(55.50% to 99.75%)	(99.45% to 100%)	(55.61% to 98.47%)	(99.37% to 99.98%)	(99.30% to 99.98%)
**Gram-Positive Bacilli**	2.24%	GRAM	82.61% (61.22% to 95.05%)	100%	100%	99.6% (99.04% to 99.84%)	99.61% (99.01% to 99.89%)
		SEM	86.96%	99.9%	95.25%	99.7%	99.61%
			(66.41% to 97.22%)	(99.45% to 100%)	(73.74% to 99.31%)	(99.15% to 99.90%)	(99.01% to 99.89%)
*Actinomyces* sp.	0.29%	SEM	100%	100%	100%	100%	100%
*Bacillus* sp.	0.78%	SEM	87.5% (47.35% to 99.68%)	100%	100%	99.9% (99.39% to 99.98%)	99.9% (99.46% to 100%)
Other Gram-positive bacilli	0.68%	SEM	100%	100%	100%	100%	100%
*Propionibacterium* sp.	0.49%	SEM	60% (14.66% to 94.73%)	99.9% (99.46% to 100%)	75.16% (27.32% to 96.06%)	99.8% (99.43% to 99.93%)	99.71% (99.15% to 99.94%)

PPV: Positive Predictive Value; NPV: Negative Predictive Value; (*) These values are dependent on the prevalence.

**Table 3:** Performance characteristics for other microbe identification using SEM vs. Gram staining with culture-based MALDI-TOF/MS identification as a reference.

Group Identified	Prevalence	Method	Sensitivity (95% CI)	Specificity (95% CI)	PPV (95% CI) *	NPV (95% CI) *	Accuracy (95% CI) *
**Yeast**	1.36%	GRAM	100%	100%	100%	100%	100%
		SEM	78.57% (49.20% to 95.34%)	100%	100%	99.71% (99.20% to 99.89%)	99.71% (99.15% to 99.94%)
**Others**	1.07%	GRAM	90.91%	99.51%	66.69%	99.9%	99.42%
			(58.72% to 99.77%)	(98.86% to 99.84%)	(45.02% to 83.04%)	(99.36% to 99.98%)	(98.74% to 99.79%)
		SEM	45.45%	99.71%	62.52%	99.41%	99.12%
			(16.75% to 76.62%)	(99.14% to 99.94%)	(31.21% to 85.98%)	(99.00% to 99.66%)	(98.35% to 99.60%)

PPV: Positive Predictive Value; NPV: Negative Predictive Value; (*) These values are dependent on the prevalence.

## Data Availability

Not applicable.

## Supplementary Materials

The following are available online at https://www.mdpi.com/article/10.3390/microorganisms9061170/s1, Table S1. Pure bacterial cultures inoculated in virgin blood culture bottles to simulate the clinical blood culture growth conditions and optimise the SEM identification strategy. Table S2. Cost calculations for sample preparation of SEM and Gram-stained slides. a. Manufacturer’s recommended number of samples per 500 mL solution bottles. Figure S1. Genus occurrence of the micro-organisms present in 1160 positive blood cultures. Figure S2. Cell-wall analysis using Image-J. Figure S3. Bacterial widths measurements performed on Image-J. Figure S4. Main issues causing discordances in the SEM identification strategy.

## References

[1] Romero-Gómez M.-P., Gómez-Gil R., Paño-Pardo J.R., Mingorance J. (2012). Identification and Susceptibility Testing of Microorganism by Direct Inoculation from Positive Blood Culture Bottles by Combining MALDI-TOF and Vitek-2 Compact Is Rapid and Effective. J. Infect..

[2] Kok J., Thomas L.C., Olma T., Chen S.C.A., Iredell J.R. (2011). Identification of Bacteria in Blood Culture Broths Using Matrix-Assisted Laser Desorption-Ionization Sepsityper^TM^ and Time of Flight Mass Spectrometry. PLoS ONE.

[3] Dubourg G., Raoult D. (2016). Emerging Methodologies for Pathogen Identification in Positive Blood Culture Testing. Expert Rev. Mol. Diagn..

[4] Dubourg G., Raoult D., Fenollar F. (2019). Emerging Methodologies for Pathogen Identification in Bloodstream Infections: An Update. Expert Rev. Mol. Diagn..

[5] Verroken A., Defourny L., Waroux O.P., de Belkhir L., Laterre P.-F., Delmée M., Glupczynski Y. (2016). Clinical Impact of MALDI-TOF MS Identification and Rapid Susceptibility Testing on Adequate Antimicrobial Treatment in Sepsis with Positive Blood Cultures. PLoS ONE.

[6] Wolk D.M., Johnson J.K. (2019). Rapid Diagnostics for Blood Cultures: Supporting Decisions for Antimicrobial Therapy and Value-Based Care. J. Appl. Lab. Med..

[7] Aoki Y. (2010). Refinement of presumptive antimicrobial therapy based on initial microbiological information on positive blood culture. Rinsho Byori.

[8] Boyanova L. (2018). Direct Gram Staining and Its Various Benefits in the Diagnosis of Bacterial Infections. Postgrad. Med..

[9] SchØNheyder H.C., HØJbjerg T. (1995). The Impact of the First Notification of Positive Blood Cultures on Antibiotic Therapy. APMIS.

[10] Barenfanger J., Graham D.R., Kolluri L., Sangwan G., Lawhorn J., Drake C.A., Verhulst S.J., Peterson R., Moja L.B., Ertmoed M.M. (2008). Decreased Mortality Associated With Prompt Gram Staining of Blood Cultures. Am. J. Clin. Pathol..

[11] Nagata K., Mino H., Yoshida S. (2010). Usefulness and limit of Gram staining smear examination. Rinsho Byori.

[12] Beveridge T.J. (1990). Mechanism of Gram Variability in Select Bacteria. J. Bacteriol..

[13] Esteban J., García-Calvo G., Jiménez-Castillo P., Soriano F. (1996). Failure of Gram Stain to Detect Propionibacterium Acnes in Specimens from Clinically Significant Infections. J. Clin. Microbiol..

[14] Pliakos E.E., Andreatos N., Shehadeh F., Ziakas P.D., Mylonakis E. (2018). The Cost-Effectiveness of Rapid Diagnostic Testing for the Diagnosis of Bloodstream Infections with or without Antimicrobial Stewardship. Clin. Microbiol. Rev..

[15] Verroken A., Despas N., Rodriguez-Villalobos H., Laterre P.-F. (2019). The Impact of a Rapid Molecular Identification Test on Positive Blood Cultures from Critically Ill with Bacteremia: A Pre-Post Intervention Study. PLoS ONE.

[16] Hou T.-Y., Chiang-Ni C., Teng S.-H. (2019). Current Status of MALDI-TOF Mass Spectrometry in Clinical Microbiology. J. Food Drug Anal..

[17] MacVane S.H., Nolte F.S. (2016). Benefits of Adding a Rapid PCR-Based Blood Culture Identification Panel to an Established Antimicrobial Stewardship Program. J. Clin. Microbiol..

[18] Mancini N., Infurnari L., Ghidoli N., Valzano G., Clementi N., Burioni R., Clementi M. (2014). Potential Impact of a Microarray-Based Nucleic Acid Assay for Rapid Detection of Gram-Negative Bacteria and Resistance Markers in Positive Blood Cultures. J. Clin. Microbiol..

[19] Peker N., Couto N., Sinha B., Rossen J.W. (2018). Diagnosis of Bloodstream Infections from Positive Blood Cultures and Directly from Blood Samples: Recent Developments in Molecular Approaches. Clin. Microbiol. Infect..

[20] Zrodlowski T., Flis A., Ziętkiewicz M., Drwiła R., Gosiewski T. (2017). Fluorescent in Situ Hybridization and Gram-stained Smears of Whole Blood as Complementary Screening Tools in the Diagnosis of Sepsis. Pol. Arch. Intern. Med..

[21] Koh H., Aimoto M., Matsuhisa A., Inoue S.-I., Katayama T., Okamura H., Yoshimura T., Koh S., Nanno S., Nishimoto M. (2016). Combinational Approach Using in Situ Hybridization Targeting 23S Ribosomal RNA Genes and Blood Cultures for Bacterial Identification in Patients with Neutropenia and Fever. J. Infect. Chemother..

[22] Berild D., Mohseni A., Diep L.M., Jensenius M., Ringertz S.H. (2006). Adjustment of Antibiotic Treatment According to the Results of Blood Cultures Leads to Decreased Antibiotic Use and Costs. J. Antimicrob. Chemother..

[23] Bourbeau P.P., Ledeboer N.A. (2013). Automation in Clinical Microbiology. J. Clin. Microbiol..

[24] Dauwalder O., Landrieve L., Laurent F., de Montclos M., Vandenesch F., Lina G. (2016). Does Bacteriology Laboratory Automation Reduce Time to Results and Increase Quality Management?. Clin. Microbiol. Infect..

[25] Le Minor L., Véron M. (1989). Bactériologie Médicale.

[26] Schindelin J., Arganda-Carreras I., Frise E., Kaynig V., Longair M., Pietzsch T., Preibisch S., Rueden C., Saalfeld S., Schmid B. (2012). Fiji: An Open-Source Platform for Biological-Image Analysis. Nat. Methods.

[27] Seng P., Drancourt M., Gouriet F., La Scola B., Fournier P.-E., Rolain J.M., Raoult D. (2009). Ongoing Revolution in Bacteriology: Routine Identification of Bacteria by Matrix-Assisted Laser Desorption Ionization Time-of-Flight Mass Spectrometry. Clin. Infect. Dis..

[28] Sogaard M., Norgaard M., Schonheyder H.C. (2007). First Notification of Positive Blood Cultures and the High Accuracy of the Gram Stain Report. J. Clin. Microbiol..

[29] Mutters N.T., Hodiamont C.J., de Jong M.D., Overmeijer H.P.J., van den Boogaard M., Visser C.E. (2014). Performance of Kiestra Total Laboratory Automation Combined with MS in Clinical Microbiology Practice. Ann. Lab. Med..

[30] Fenollar F., Raoult D. (2000). Comparison of a Commercial Disk Test with Vancomycin and Colimycin Susceptibility Testing for Identification of Bacteria with Abnormal Gram Staining Reactions. EJCMID.

[31] Haddad G., Bellali S., Fontanini A., Francis R., La Scola B., Levasseur A., Bou Khalil J., Raoult D. (2020). Rapid Scanning Electron Microscopy Detection and Sequencing of Severe Acute Respiratory Syndrome Coronavirus 2 and Other Respiratory Viruses. Front. Microbiol..

[32] Haddad G., Fontanini A., Bellali S., Takakura T., Ominami Y., Hisada A., Hadjadj L., Rolain J.M., Raoult D., Bou Khalil J. (2021). Rapid Detection of Imipenem Resistance in Gram-Negative Bacteria Using Tabletop Scanning Electron Microscopy: A Preliminary Evaluation. Front. Microbiol..

[33] Hannachi N., Lepidi H., Fontanini A., Takakura T., Bou-Khalil J., Gouriet F., Habib G., Raoult D., Camoin-Jau L., Baudoin J.-P. (2020). A Novel Approach for Detecting Unique Variations among Infectious Bacterial Species in Endocarditic Cardiac Valve Vegetation. Cells.

[34] Verroken A., Defourny L., Lechgar L., Magnette A., Delmée M., Glupczynski Y. (2015). Reducing Time to Identification of Positive Blood Cultures with MALDI-TOF MS Analysis after a 5-h Subculture. Eur. J. Clin. Microbiol. Infect. Dis..

[35] Gaibani P., Rossini G., Ambretti S., Gelsomino F., Pierro A.M., Varani S., Paolucci M., Landini M.P., Sambri V. (2009). Blood Culture Systems: Rapid Detection—How and Why?. Int. J. Antimicrob. Agents.

