# Supplementary material for: Scanning Electron Microscope: A New Potential Tool to Replace Gram Staining for Microbe Identification in Blood Cultures
Source: Microorganisms. 2021 May 28;9(6):1170. doi: 10.3390/microorganisms9061170 (PMC8227564; doi:10.3390/microorganisms9061170)
Supplement: Supplementary file 1 [file microorganisms-09-01170-s001.zip › microorganisms-1243035-Supplementary material.pdf]

Supplementary Data

Table S1. Pure bacterial cultures inoculated in virgin blood culture bottles to simulate the clinical blood culture growth conditions and optimize the SEM identification strategy.

Pure culture isolates		
Acinetobacter junii	*Acinetobacter timonensis*	*Morganella morganii*
Acinetobacter baumannii	*Acinetobacter trogloensis*	*Proteus hauseri*
Acinetobacter baylyi	*Acinetobacter ursingii*	*Proteus mirabilis*
Acinetobacter bereziniae	*Acinetobacter variabilis*	*Proteus penneri*
Acinetobacter calcoaceticus	*Acinetobacter septicus*	*Proteus rettgeri*
Acinetobacter courvalinii	*Citrobacter amalonaticus*	*Proteus stuartii*
Acinetobacter gouillouiae	*Citrobacter brakii*	*Proteus vulgaris*
Acinetobacter haemolyticus	*Citrobacter farmeri*	*Serratia marcescens*
Acinetobacter indicus	*Citrobacter freudii*	*Shigella flexneri*
Acinetobacter jenensis	*Citrobacter koseri*	*Clostridium difficile*
Acinetobacter johnsonii	*Enterobacter cloacae*	*Brevibacterium casei*
Acinetobacter kempfi	*Escherichia albertii*	*Propionibacterium acnes*
Acinetobacter lwoffi	*Escherichia coli*	*Bacillus cereus*
Acinetobacter nosocomialis	*Escherichia fergusonii*	*Staphylococcus aureus*
Acinetobacter parvus	*Escherichia hermannii*	*Staphylococcus hominis*
Acinetobacter pittii	*Escherichia vulneris*	*Streptococcus anginosus*
Acinetobacter radioresistens	*Klebsiella aerogenes*	*Enterococcus faecalis*
Acinetobacter schindleri	*Klebsiella oxytoca*	*Neisseria meningitidis*
Acinetobacter mediterraneensis	*Klebsiella pneumoniae*	*Moraxella catarrhalis*
	Klebsiella variicola	*Neisseria gonorrhoeae*

Table S2. Cost calculations for sample preparation of SEM and Gram-stained slides.

Sample preparation for SEM	Bulk quantity	Bulk cost	Number of samples /bulk	Cost for 12 samples	Cost for one sample
**PTA**	25 g	46.50 €	100,000	0.005 €	0.0004 €
**Poly-L-lysine**	50 mL	126.00 €	5,000	0.302 €	0.0252 €
**12-well slides**	1,000	525.97 €	12,000	0.525 €	0.0438 €
**Total**				**0.832 €**	**0.0695 €**

Sample preparation for AeroSpray Gram staining	Bulk quantity	Bulk cost	Number of samples /bulk	Cost for 12 samples	Cost for one sample
**Cristal violet**	500 mL	21.64 €	1,032 [a]	0.251 €	0.0209 €
**Iodine**	500 mL	21.82 €	996 [a]	0.262 €	0.0219 €
**Safranine**	500 mL	37.03 €	1,032 [a]	0.430 €	0.0358 €
**Wash solution**	500 mL	31.68 €	1,032 [a]	0.368 €	0.0307 €
**Glass slides**	50	4.47 €	50	1.072 €	0.0894 €
**Total**				**2.387 €**	**0.1988 €**

[a] Manufacturer's recommended number of samples per 500 mL solution bottles.

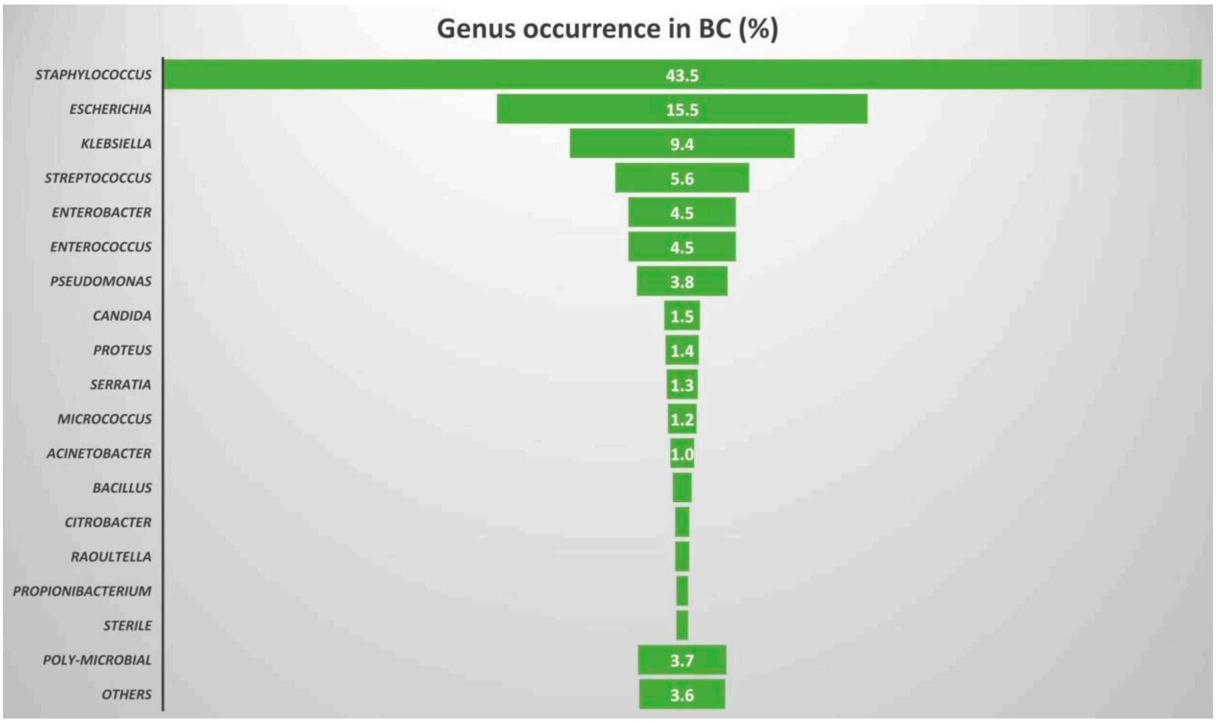

Figure S1. Genus occurrence of the microorganisms present in 1160 positive blood cultures.

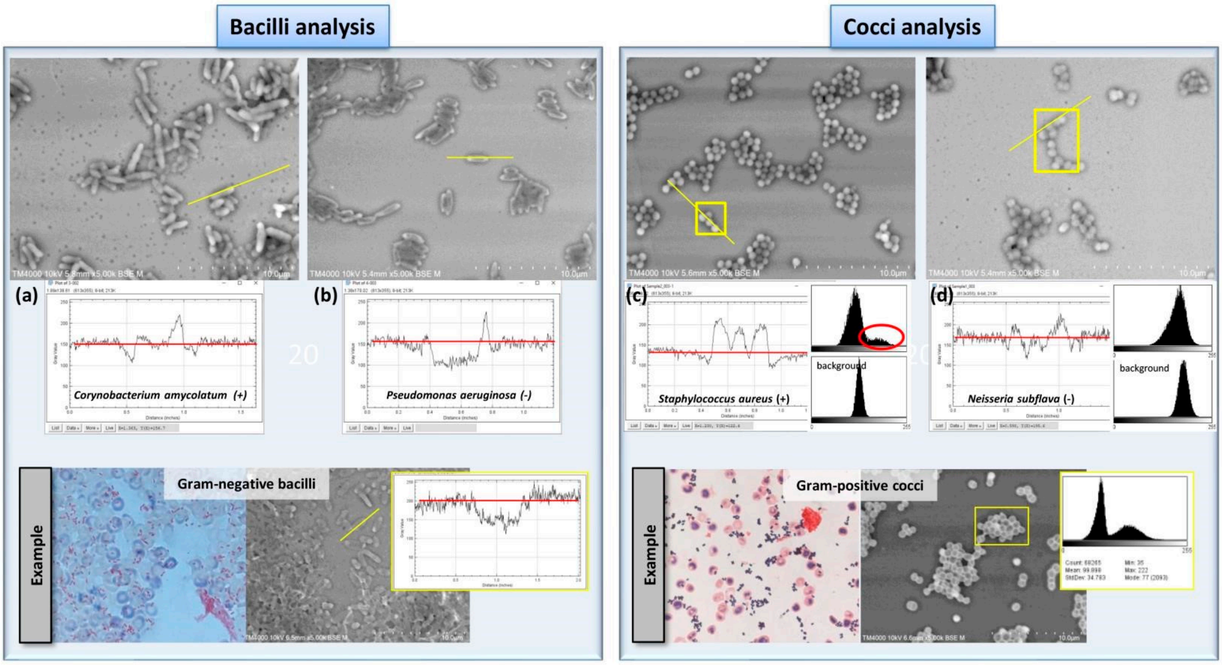

Figure S2. Cell-wall analysis using Image-J. When analyzing bacilli, we compare the brightness in the middle part of the microbe to that of the background. When the analyzed signal is brighter than the background as observed on the grayscale line profile **(a),** it is judged as Gram-positive. If the middle part is darker **(b)**, we judge it as Gram-negative. For the cocci analysis, the same strategy was applied either on the grayscale line profile or the grayscale histogram profile, showing in both cases a bright signal in the case of Gram-positive cocci **(c)** that is not detected in the Gram-negative cocci profile **(d)**

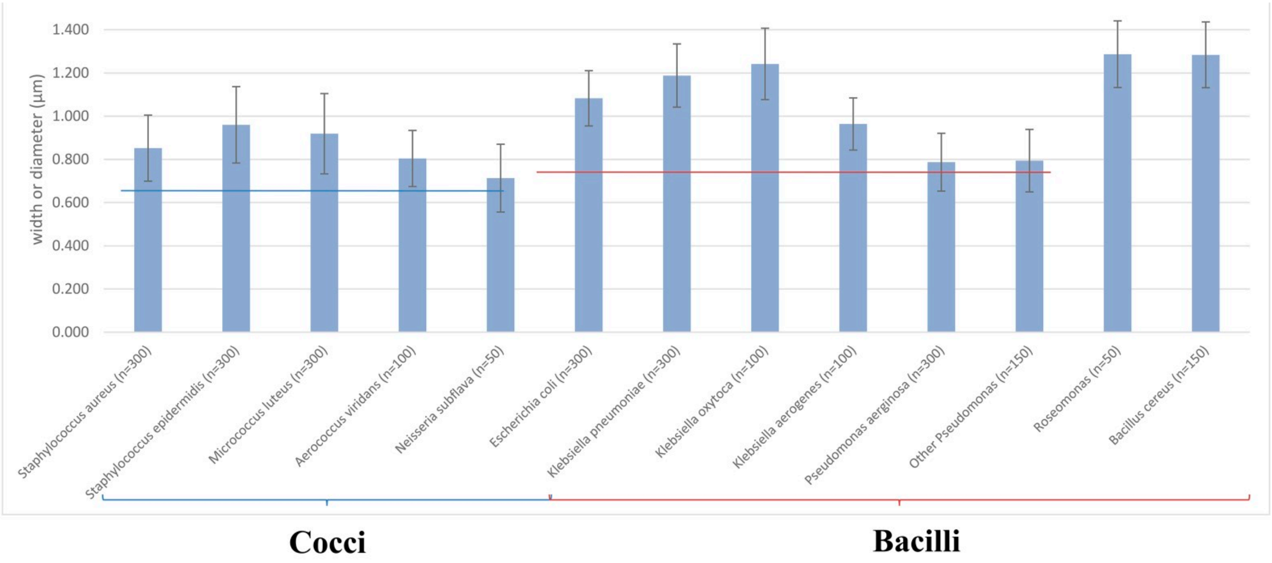

Figure S3. Bacterial widths measurements realized on Image-J. 50 to 300 bacteria were measured by species.

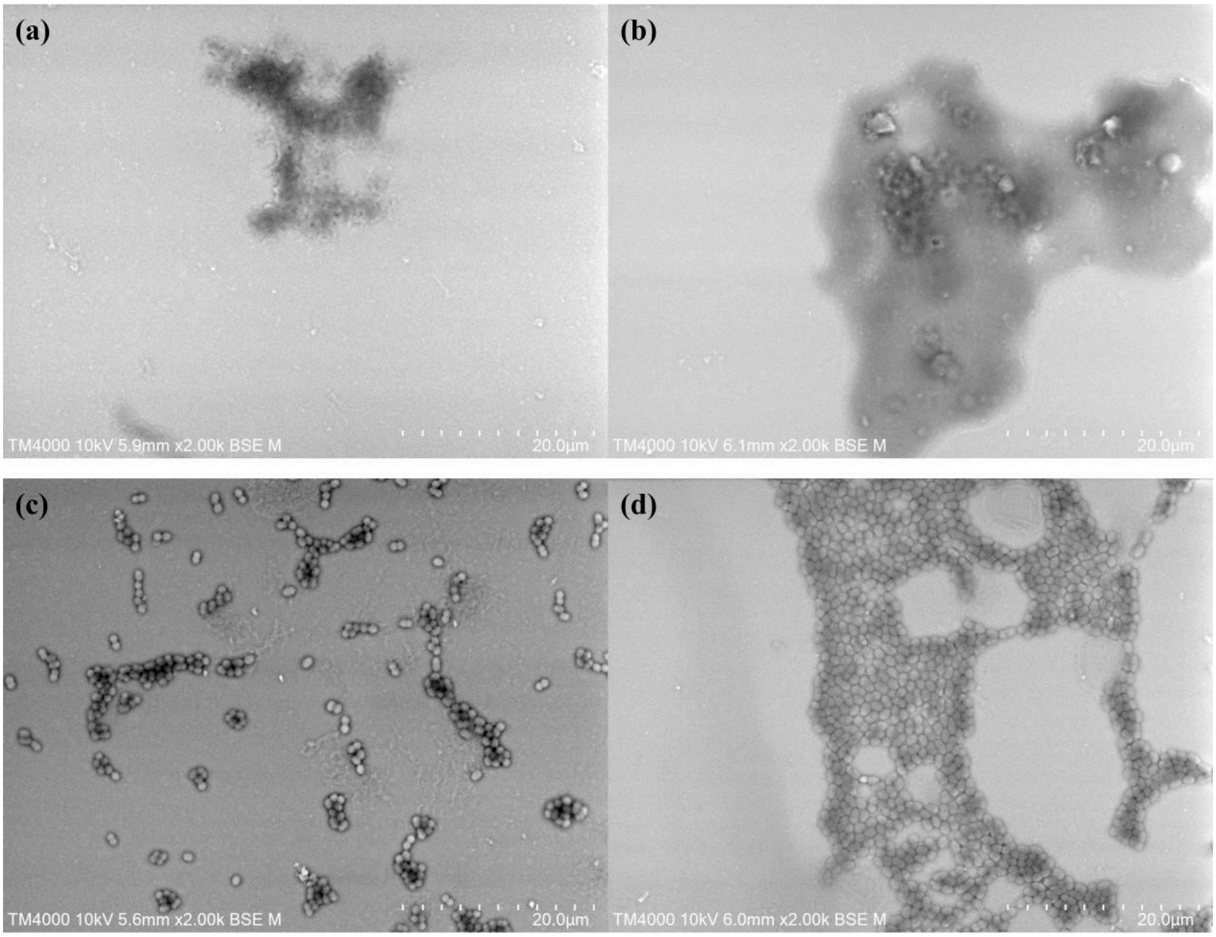

Figure S4. Main issues causing discordances in the SEM identification strategy. Blood cultures positive for *Candida parapsilosis* **(a)** and *Streptococcus australis* **(b)** according to MALD-TOF/MS with no visible microbes by SEM. Blood cultures positive for *Streptococcus salivarius* **(c)** and *Enterococcus faecalis* **(d)** presenting a cluster cocci arrangement.
